# The duct of von Ebner’s glands is a source of *Sox10* ^+^ taste bud progenitors and susceptible to pathogen infections

**DOI:** 10.3389/fcell.2024.1460669

**Published:** 2024-08-23

**Authors:** Wenxin Yu, Maria Eleni Kastriti, Mohamed Ishan, Saurav Kumar Choudhary, Md Mamunur Rashid, Naomi Kramer, Hy Gia Truong Do, Zhonghou Wang, Ting Xu, Robert F. Schwabe, Kaixiong Ye, Igor Adameyko, Hong-Xiang Liu

**Affiliations:** ^1^ Department of Animal and Dairy Science, Regenerative Bioscience Center, College of Agricultural and Environmental Sciences, University of Georgia, Athens, GA, United States; ^2^ Department of Neuroimmunology, Medical University of Vienna, Vienna, Austria; ^3^ Institute of Bioinformatics, University of Georgia, Athens, GA, United States; ^4^ Department of Genetics, University of Georgia, Athens, GA, United States; ^5^ Department of Medicine, College of Physicians and Surgeons, Columbia University, New York, NY, United States; ^6^ Department of Physiology and Pharmacology, Karolinska Institutet, Solna, Sweden

**Keywords:** taste buds, progenitor, *Sox10*, von Ebner’s glands, connective tissue, single cell RNA-sequencing, cell differentiation, type-III taste cell

## Abstract

**Introduction:**

We have recently demonstrated that *Sox10*-expressing (*Sox10* ^+^) cells give rise to mainly type-III neuronal taste bud cells that are responsible for sour and salt taste. The two tissue compartments containing *Sox10* ^+^ cells in the surrounding of taste buds include the connective tissue core of taste papillae and von Ebner’s glands (vEGs) that are connected to the trench of circumvallate and foliate papillae.

**Methods:**

In this study, we performed single cell RNA-sequencing of the epithelium of *Sox10-Cre/tdT* mouse circumvallate/vEG complex and used inducible Cre mouse models to map the cell lineages of vEGs and/or connective tissue (including stromal and Schwann cells).

**Results:**

Transcriptomic analysis indicated that *Sox10* expression was enriched in the cell clusters of vEG ducts that contained abundant proliferating cells, while *Sox10-Cre/tdT* expression was enriched in type-III taste bud cells and vEG ductal cells. *In vivo* lineage mapping showed that the traced cells were distributed in circumvallate taste buds concurrently with those in the vEGs, but not in the connective tissue. Moreover, multiple genes encoding pathogen receptors were enriched in the vEG ducts hosting *Sox10* ^+^ cells.

**Discussion:**

Our data supports that it is the vEGs, not connective tissue core, that serve as the niche of *Sox10* ^+^ taste bud progenitors. If this is also true in humans, our data indicates that vEG duct is a source of *Sox10* ^+^ taste bud progenitors and susceptible to pathogen infections.

## Introduction

Taste bud cells have a short lifespan and need to be constantly replaced (Beidler and Smallman, 1965; Conger and Wells, 1969; Cohn et al., 2010; Perea-Martinez et al., 2013; Miura et al., 2014). Thus, stem/progenitor cells in the surrounding tissue compartments are essential for taste bud homeostasis. It has been well documented that the *Krt5/14* ^+^ (Okubo et al., 2009), *P63* ^+^ (Okubo et al., 2009), *Sox2* ^+^ (Okubo et al., 2006; Okubo et al., 2009; Arnold et al., 2011; Ohmoto et al., 2017; Castillo-Azofeifa et al., 2018; Ohmoto et al., 2020; Ohmoto et al., 2022), *Lgr5* ^+^ (Takeda et al., 2013; Yee et al., 2013; Ren et al., 2014), and *Gli1* ^+^ (Liu et al., 2013) basal cells in taste bud-surrounding lingual epithelium are taste bud progenitors. We have recently found that cells expressing SRY-related HMG-box gene 10 (*Sox10* ^+^) give rise to taste bud cells that are mainly type-III with low levels of Krt8 (Yu et al., 2020) that has been regarded as a pan-taste cell marker. Using *in situ* hybridization for *Sox10*, we identified two tissue compartments under lingual epithelium that contain *Sox10* ^+^ cells, i.e., connective tissue core and von Ebner’s glands (vEGs) (Yu et al., 2020) -- minor salivary glands connected to the trench of circumvallate and foliate papillae (Hand et al., 1999; Yu et al., 2020). The connective tissue core and/or vEGs may represent a novel source of progenitors for taste buds.

In our recent study to map the lineage of neural crest exclusively, we found that *Sox10-iCreER/tdT* ^*Tmx@E7.5*^ traced cells were present in underlying connective tissue but not in taste buds (Yu et al., 2021a), which indicates that taste bud cells do not originate from neural crest and the derived connective tissue. However, the questions remain where and what cells in non-neural crest-derived connective tissue and/or endodermal vEGs (Baumgartner, 1917; Batsakis, 1980; Marks and Cahill, 1987) are progenitor cells for taste buds.

In this study, we used single cell transcriptomic analyses on the epithelium of circumvallate/vEG complex and multiple inducible Cre mouse models to map the lineage of cells surrounding circumvallate taste buds including the cells in the connective tissue core and the cells in vEGs. Our data indicates that *Sox10* ^+^ cells in the duct of vEGs, but not in the connective tissue core of taste papillae, serve as progenitors for taste buds without being transited to basal cells in the stratified tongue epithelium. Moreover, the *Sox10* ^+^ vEG ducts enrich multiple genes encoding pathogen receptors indicating its potential susceptibility to pathogen infections.

## Results

### Single cell transcriptomic analyses reveal *Sox10* expression in vEG ducts and *Sox10-Cre/tdT* expression in Type-III taste cells

**FIGURE 1:**
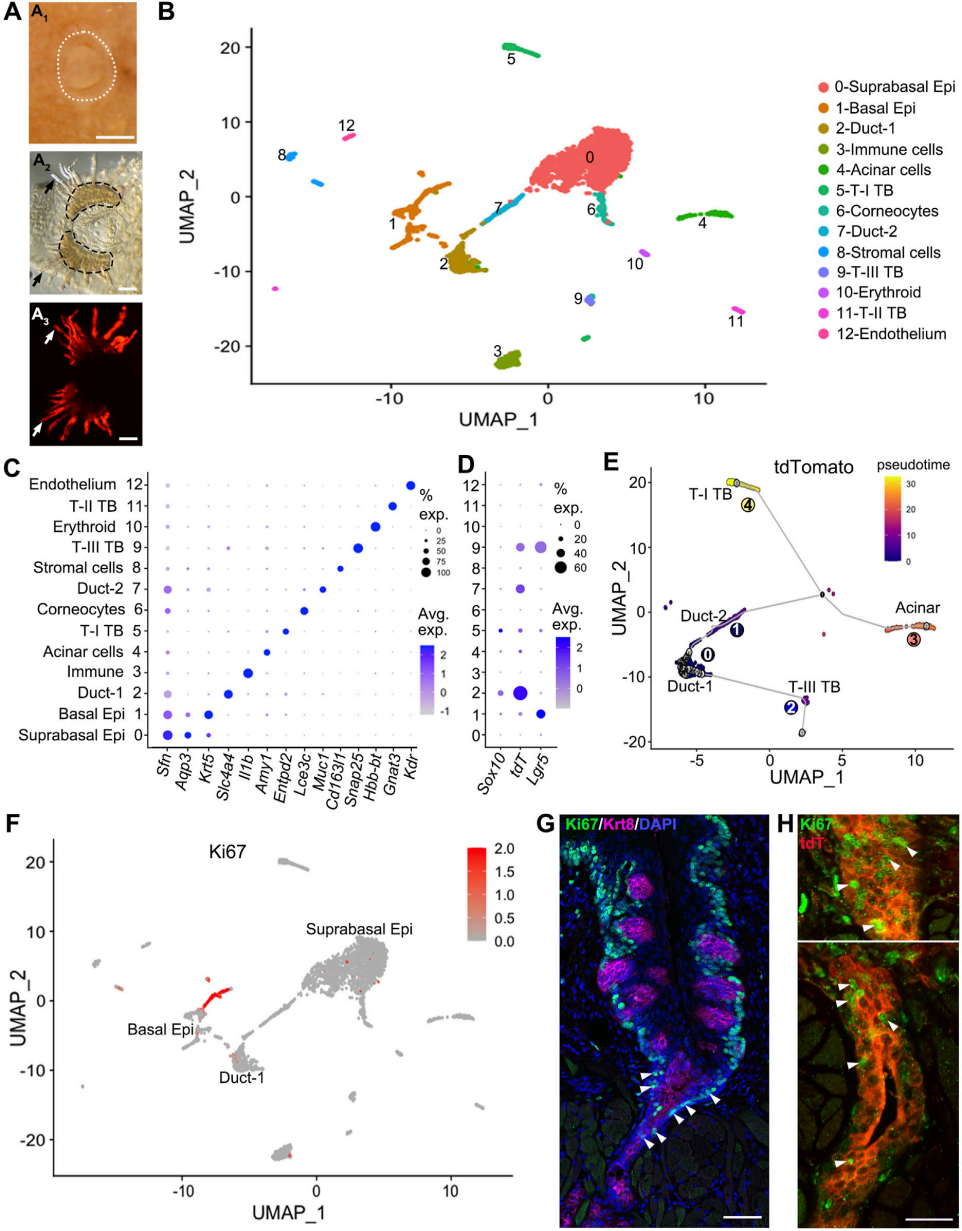
*Sox10* expression is enriched in the Duct-1 of vEGs while *Sox10-Cre/tdT* marks type-III taste bud and Duct-2 cells. **(A)** whole mount images showing the circumvallate papilla and peeled epithelial sheet of circumvallate/vEG complex for cell dissociation. White dotted lines (A_1_) encircle the circumvallate papilla in postnatal tongue. Black dashed lines (A_2_) encircle the trenches of circumvallate papilla. Dark arrows (A_2_) and white arrows (A_3_) point to the vEG ducts. Epi: epithelium; T-I, T-II, T-III: type-I, II, III; TB: taste bud. **(B)** t-SNE map to illustrate the 13 major cell clusters identified in the scRNA-seq data from target tissues shown in **(A)**. **(C)** Dot plot to illustrate the expression of typical markers for identifying the cell clusters. **(D)** Dot plot to illustrate gene expression of *Sox10, tdT, Lgr5* across cell clusters. **(E)** Pseudotime analysis on the t-SNE map by setting the Duct-1 as root illustrates the cell position along the trajectory. **(F)** Feature plots of *Ki67* transcripts on the UMAP showing cell cluster expressing *Ki67*. **(G,H)** Single-plane laser scanning confocal photomicrographs of coronal sections of circumvallate papilla to demonstrate the distribution of Ki67^+^ cells (green) in wild type (G) and *Sox10-Cre/tdT* **(H)** mice. Taste buds are marked by Krt8 immunosignals [(G), magenta]. White Arrowheads in **(G)** point to Ki67^+^ immunostained cells in the transition area between trench of circumvallate papilla and vEG duct. White arrowheads in **(H)** point to selected Ki67^+^ cells in the opening area (upper panel) and deeper layer (lower panel) of vEG ducts. Scale bars: 250 μm in A_1_; 100 μm in A_2_, A_3_; 50 μm in G; 25 μm in **(H)**.

To identify the epithelial cell population of *Sox10* ^*+*^ cells and derived cells, single cell RNA-sequencing (scRNA-Seq) was performed using dissociated cells from the epithelial sheets of the circumvallate papilla/vEG complex in *Sox10-Cre/tdT* mice (Figure 1A_1–3_). *Sox10-Cre/tdT*-traced signals were robust in the vEG ducts that are connected to the trench of circumvallate papilla (Figure 1A_3_). At least 13 cell clusters were identified according to their associated signature genes (Figure 1B). Typical markers were used for identifying the 13 major cell clusters (Figure 1C). Type I-III taste bud cells were distinctly identifiable (*Entpd2* ^+^ type-I, *Gnat3* ^+^ type-II, *Snap25* ^+^ type-III). Cell types existing in the stratified epithelium were found including the basal (*Krt5* ^+^), supra-basal (*Sfn* ^+^ *Aqp3* ^+^) and superficial (corneocytes) (*Lce3c* ^+^) layers of epithelial cells. Three clusters of glandular cells were detected including two of ductal cells (*Slc4a4* ^+^ Duct-1, *Muc1* ^+^ Duct-2) and *Amy1* ^+^ acinar cells. In addition, a few other small clusters were identified representing erythrocytes (*Hbb*-bt^+^), immune cells (*Il1b* ^+^), and stromal cells (*Cd163l1* ^+^ *Vim* ^+^).

Among these cell clusters, *Sox10* expression was enriched in the Duct-1 of vEGs and a small proportion of differentiated type-I taste bud cells (Figure 1D). In contrast, *tdT* expression marking the *Sox10* ^+^ cell lineage was enriched in type-III taste bud cells and Duct-2 of vEGs in which *Sox10* expression was not detected. Moreover, *tdT* expression was detected at a high level in over 60% of the Duct-1 cells. Of note, *Lgr5*, the well-known taste bud stem cell marker, was expressed in basal epithelial cells (e.g., known progenitors for taste buds) and type-III taste bud cells, but not detected in any types of vEG cells.

The differentiation trajectory of *Sox10-Cre/tdT* ^+^ cells with pseudotime analysis indicated an order of cell clusters from the vEG Duct-1 to the two immediate neighbor cell compartments, i.e., Duct-2 and taste bud (mainly type-III) cells (Figure 1E). The two clusters of further order of cells include acinar and type-I taste bud cells that express *Sox10* in themselves.

To identify the proliferating and potential progenitors for taste buds, enrichment of *Ki67* expression was found in basal and supra-basal epithelial cells, as well as in the ducts of vEGs (Figure 1F). Immunosignals of Ki67 confirmed the abundant distribution of Ki67^+^ proliferating cells in the ducts of vEGs in addition to the basal and some supra-basal epithelial cells surrounding taste buds (Figure 1G). These Ki67^+^ vEG ductal cells were largely *Sox10-Cre/tdT* ^+^ cells (Figure 1H).

### *Sox10-iCreER* ^*T2*^ */tdT* and *Sox10-CreER* ^*T2*^ */YFP* concurrently marks von Ebner’s glands and circumvallate taste buds

**FIGURE 2:**
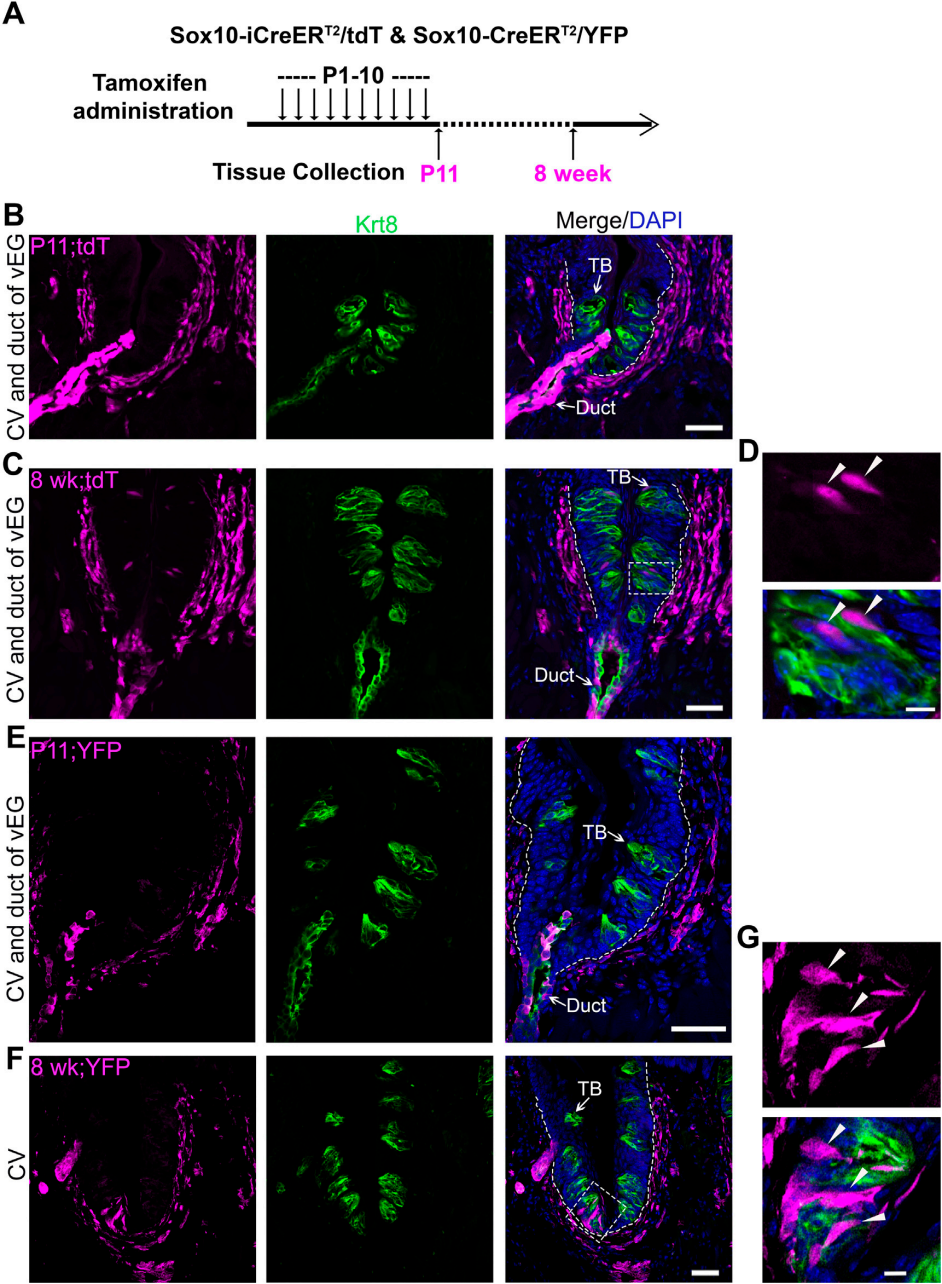
Cell labeling and lineage mapping of *Sox10* ^+^ cells in von Ebner’s glands and circumvallate taste buds. **(A)** A schematic diagram to show the experimental paradigm in *Sox10-iCreER* ^*T2*^ */tdT* and *Sox10-CreER/YFP* mice. **(B–G)** Single-plane laser scanning confocal photomicrographs on coronal sections of circumvallate papilla. The distribution of *Sox10-iCreER* ^*T2*^ */tdT* **(B–D)** and *Sox10-CreER/YFP*-traced **(E–G)** cells (magenta) of P11 **(B,E)** and 8-week-old **(C,F)** mice with tamoxifen administration from P1 to P10. **(D,G)** High-power images of the area enclosed in the dashed square in **(C,F)**. Arrows point to Krt8^+^ immunostained (green) cells in taste buds (TB) and ductal (Duct) cells of von Ebner’s gland. Arrowheads in **(D,G)** point to the tdT^+^ and YFP^+^ cells in taste buds. White dashed lines mark the borders between the epithelium and surrounding connective tissue. Scale bars: 50 μm in **(B,C,E,F)**, 10 μm in **(D,G)**.

To verify the findings in transcriptomic analyses, inducible Cre transgenic mouse models *Sox10-iCreER* ^*T2*^ */tdT* and *Sox10-CreER* ^*T2*^ */YFP* were used to evaluate the distribution of *Sox10* ^+^ cell lineage in circumvallate taste buds and its association with that in vEGs. Our previous study has ruled out the derivation of taste buds from *Sox10* ^+^ cells in the neural crest with early induction of Cre in embryos (Yu et al., 2021a). Thus, we initiated *in vivo* studies with daily tamoxifen administration from P1 to P10 (Figure 2A; Supplementary Figure 1A), we observed that at P11, vEGs (Figures 2B, E; Supplementary Figure 1B) and connective tissue (Figures 2B, E) were abundantly labeled. At this early time point tdT^+^/YFP^+^ cells were not seen in circumvallate taste buds nor the taste bud-surrounding lingual epithelial cells (Figures 2B, E). When given a period of approximately 7 weeks after the termination of tamoxifen treatments, tdT^+^ and YFP^+^ cells were seen, albeit infrequently, in circumvallate taste buds (Figures 2C, D, F, G) in both *Sox10-iCreER* ^*T2*^ */tdT* ^Tmx@P1-10d^ and *Sox10-CreER* ^*T2*^ */YFP* ^Tmx@P1-10d^ mice. Concurrently, tdT^+^ cells sustained in vEGs (Figure 2C; Supplementary Figure 1C).

**FIGURE 3:**
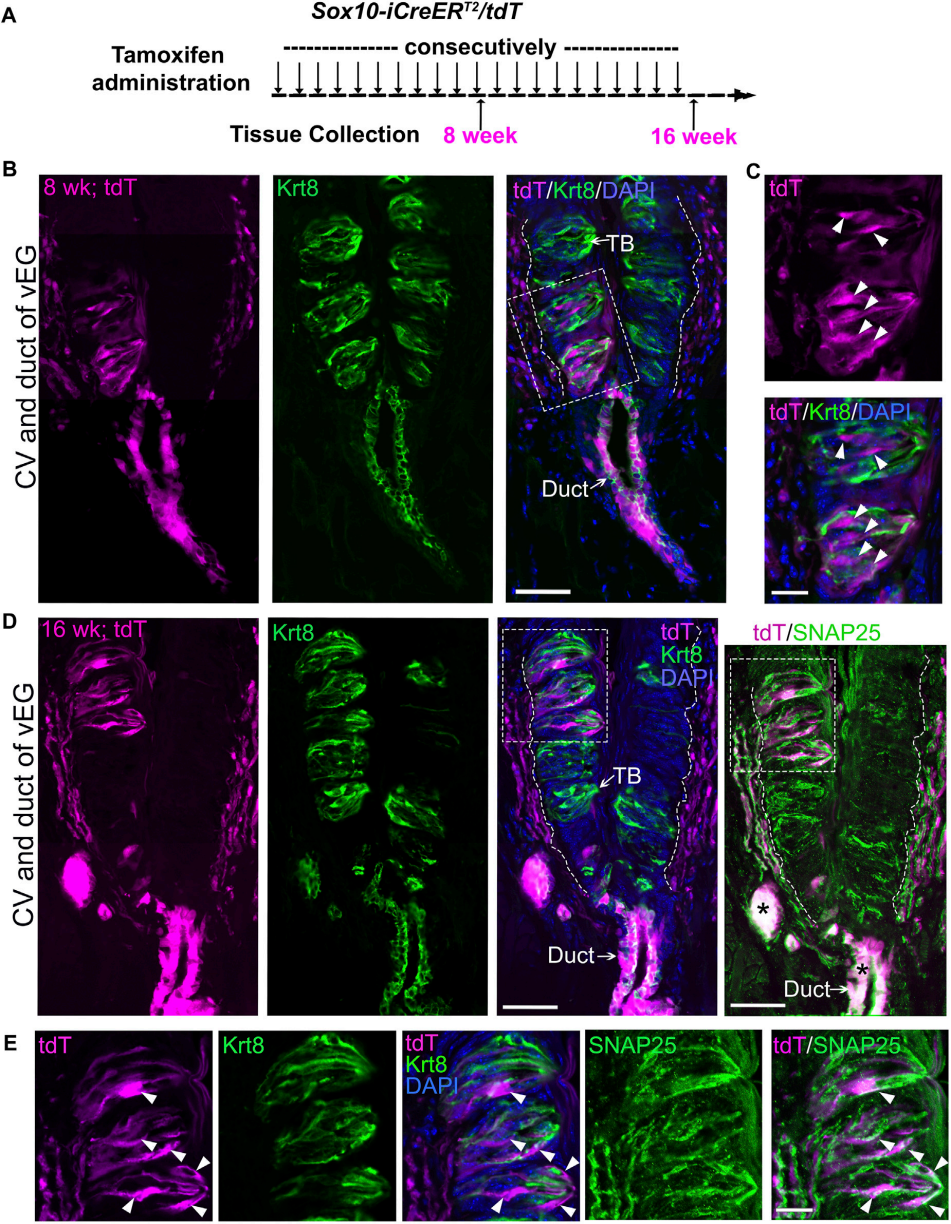
Long-term (8-week or 16-week) tracing leads to abundant distribution of *Sox10-iCreER* ^*T2*^ */tdT*-labeled cells in circumvallate taste buds concurrently with those in von Ebner’s glands. **(A)** A schematic diagram to illustrate the experimental paradigm for tamoxifen administration and tissue collection from *Sox10-iCreER* ^*T2*^ */tdT* mice at 8 or 16 weeks. **(B–E)** Single-plane laser scanning confocal images of a coronal circumvallate papilla section at 8-week **(B,C)** or 16-week **(D,E)**. tdT^+^ cells (magenta) were concurrently distributed in circumvallate taste buds and vEGs in addition to that in connective tissue. **(C,E)** are high-power images from the area enclosed in the dashed square shown in **(B,D)** respectively. Tissue sections were immunostained for pan-taste cell marker Krt8 (green) and type-III taste cell marker SNAP25 (green pseudo-color, different fluorophore from Krt8 for immunostaining). Arrows point to Krt8^+^ immunostained (green) taste buds (TB) and vEG ducts (Duct). Arrowheads in **(C,E)** point to the tdT^+^ cells in taste buds. White dashed lines in **(B,D)** mark the borders between the epithelium and surrounding connective tissue. Asterisks mark the bright tdT signals in vEG ducts bleeding-through to the GFP channel for SNAP25. Scale bars: 50 μm in **(B,D)**; 20 μm in **(C,E)**.

To increase the number of *Sox10-iCreER* ^*T2*^ */tdT* traced taste bud cells, consecutive tamoxifen administration was performed in *Sox10-iCreER* ^*T2*^ */tdT* mice from P1 day to 8 weeks or up to 16 weeks (Figure 3A; Supplementary Figure 2A). Indeed, after 8 weeks of tamoxifen treatment, *Sox10-iCreER* ^*T2*^ */tdT*-traced cells were abundantly distributed within circumvallate taste buds (Figures 3B, C) and concurrently in vEG ducts (Figure 3B) and acini (Supplementary Figure 2B). The traced taste bud cells depicted the typical fusiform shape of differentiated taste cells (Figure 3C). A similar labeling pattern was observed in *Sox10-iCreER* ^*T2*^ */tdT* mice treated with tamoxifen for 16 weeks from birth (Figures 3D, E; Supplementary Figure 2C). The tdT^+^ traced cells were positive for type-III taste bud cell marker SNAP25 while having a low level of Krt8 (Figures 3D, E). Of note, *Sox10-iCreER* ^*T2*^ */tdT*-traced cells were not observed in taste bud-surrounding lingual epithelium in all stages examined (Figures 3B, D).

### Tracing of connective tissue cell lineages does not mark circumvallate taste buds nor von Ebner’s glands (vEGs)

*Sox10-iCreER* ^*T2*^ */tdT* and *Sox10-CreER* ^*T2*^ */YFP* traced tdT^+^/YFP^+^ cells were distributed in tongue connective tissue with postnatal Cre induction (Figures 2, 3), which raise a question whether it is the *Sox10* ^+^ connective tissue cells that migrate and differentiate to taste bud cells as the previous studies suggested (Boggs et al., 2016; Yu et al., 2020). The connective tissue cells in the tongue mesenchyme and connective tissue are predominantly derived from the neural crest that does not give rise to taste buds (O’Rahilly and Müller, 2007; Liu et al., 2012; Boggs et al., 2016; Yu et al., 2020; Yu et al., 2021a). However, it is unclear how many non-neural crest-derived cells are present in the connective tissue core of taste papillae and whether these cells give rise to taste bud cells. Our recently published data showed that a single-dose tamoxifen to a pregnant dam carrying E7.5 *Sox10-iCreER* ^*T2*^ */tdT* embryos map cells extensively in the lamina propria of the mouse tongue at 8 weeks of stage (Yu et al., 2021a). In the present study, quantification of the connective tissue cells in the core of circumvallate papilla in adult *Sox10-iCreER* ^*T2*^ */tdT* ^Tmx@E7.5^ mice (Supplementary Figures 3A–C) revealed that the percentage of *Sox10-iCreER* ^*T2*^ */tdT*-traced cells versus total cells in the defined area of connective tissue was 79.94% ± 3.39% (X ± SD). Of note, tdT^+^ cells were neither found in the ducts (Supplementary Figure 3B) and acini (Supplementary Figure 4) of vEGs, nor in circumvallate taste buds marked by Krt8 immunosignals (Supplementary Figure 3B). Moreover, tdT^+^ cells were not observed in the taste bud-surrounding basal tongue epithelial cells (Supplementary Figure 3B) that are known as taste bud progenitors (Stone et al., 1995; Hamamichi et al., 2006; Okubo et al., 2009; Cohn et al., 2010; Miura and Barlow, 2010; Nguyen and Barlow, 2010; Sullivan et al., 2010).

This data suggests that up to 20% of the connective tissue cells were not traced by *Sox10-iCreER* ^*T2*^ */tdT* ^Tmx@E7.5^. This data keeps the possibility of *Sox10* ^+^ connective tissue cells as taste bud progenitors an open question warranting further studies.

In the connective tissue core, we observed two abundant and largely distinct subpopulations of cells that express either Proteolipid protein 1 (*Plp1*) (Supplementary Figure 3C), an intrinsic membrane protein specifically expressed in Schwann cells in the peripheral nervous system (Kamholz et al., 1992), or Vimentin (O’Rahilly and Müller, 2007; Liu et al., 2012; Boggs et al., 2016; Yu et al., 2020; Yu et al., 2021a), an intermediate filament protein widely used as a marker for mesenchymal stromal cells (Hol and Capetanaki, 2017). To verify the potential contribution of connective tissue cells to taste buds, we used inducible Cre mouse lines in which CreER is under the control of the promoter of *Plp1* or *Vimentin*.

**FIGURE 4:**
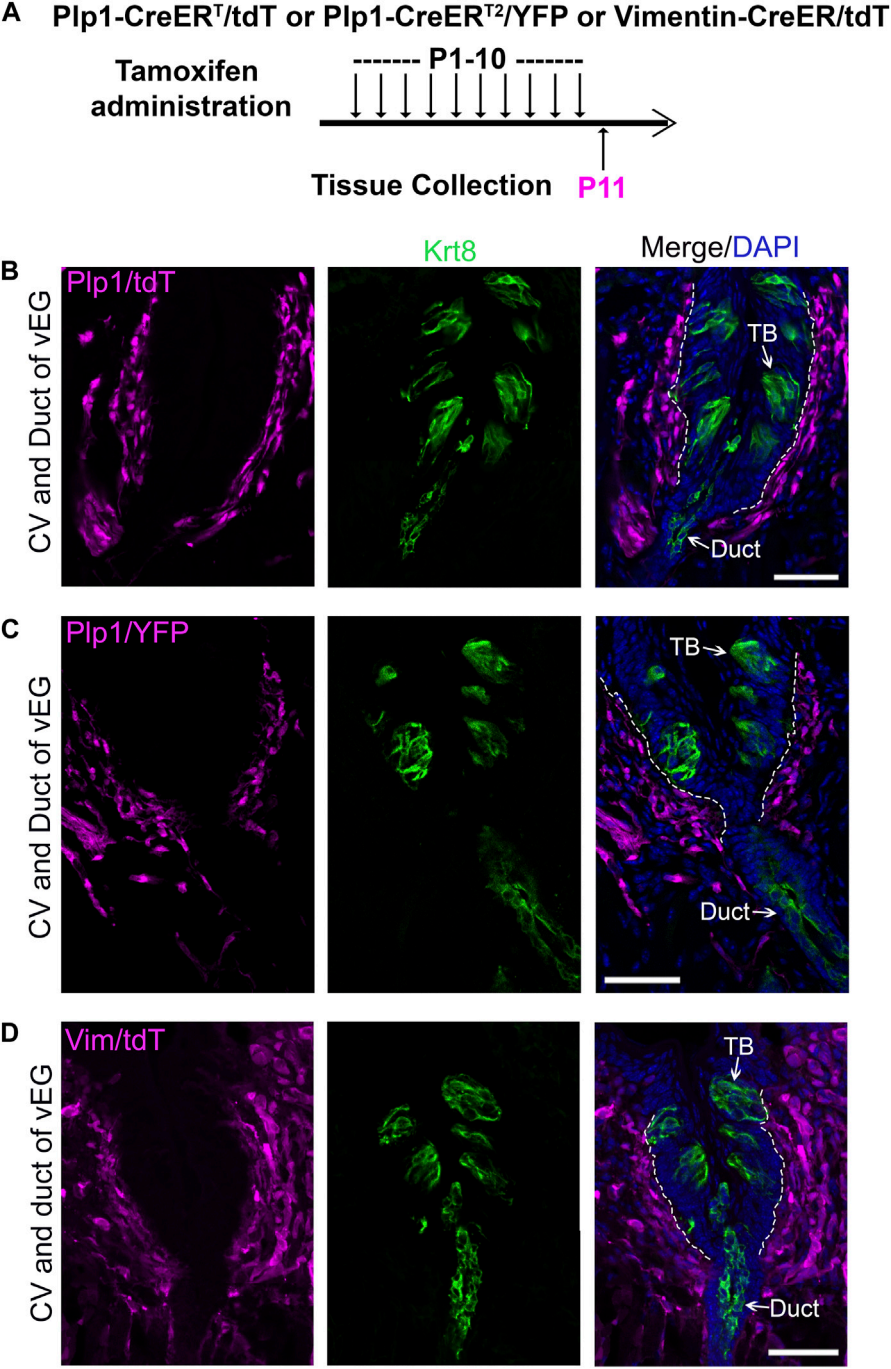
Tamoxifen administration from P1 to P10 efficiently induce labeling of connective tissue cells in the circumvallate papilla using *Plp1-CreER* ^*T*^ */tdT* or *YFP* mice (Schwann cells) and *Vimentin-CreER/tdT* mice (stromal cells). **(A)** A schematic diagram to illustrate the timeline of daily tamoxifen administration from P1-10 and tissue collection at P11. **(B–D)**: Single-plane laser scanning confocal images of circumvallate papilla and von Ebner’s gland on coronal sections of *Plp1-CreER* ^*T*^ */tdT* **(B)**, *Plp1-CreER/YFP* **(C)** and *Vimentin-CreER/tdT* **(D)** mice. Traced cells, seen as tdT^+^ **(B,D)** and YFP^+^ **(C)** cells (magenta), are abundant in the connective tissue core of circumvallate papilla. Arrows point to Krt8^+^ immunostained (green) cells in taste buds (TB), ductal (Duct) cells of von Ebner’s gland. White dashed lines mark the borders between the epithelium and surrounding connective tissue. Scale bars: 50 μm in all images.

In the P11 *Plp1-CreER* ^*T*^ */tdT* ^Tmx@P1-10d^ (Figures 4A, B), *Plp1-CreER* ^*T2*^ */YFP* ^Tmx@P1-10d^ (Figures 4A, C), and *Vimentin-CreER/tdT* ^Tmx@P1-10d^ (Figures 4A, D) mice, tdT^+^/YFP^+^ cells were abundantly marked in the connective tissue core of circumvallate papilla after daily tamoxifen treatments from P1 to P10. At this time point, no tdT^+^/YFP^+^ cells were found in taste buds and vEGs (Figures 4B–D; Supplementary Figures 5B, 5C). This data demonstrated that our tamoxifen treatment paradigm was sufficient to induce DNA recombination for tracing *Plp1* ^+^ and *Vimentin* ^+^ cell lineages in the connective tissue. The absence of tdT^+^/YFP^+^ cells in taste buds at this early time point makes these mouse models eligible for taste bud cell derivation assay at later time points.

**FIGURE 5:**
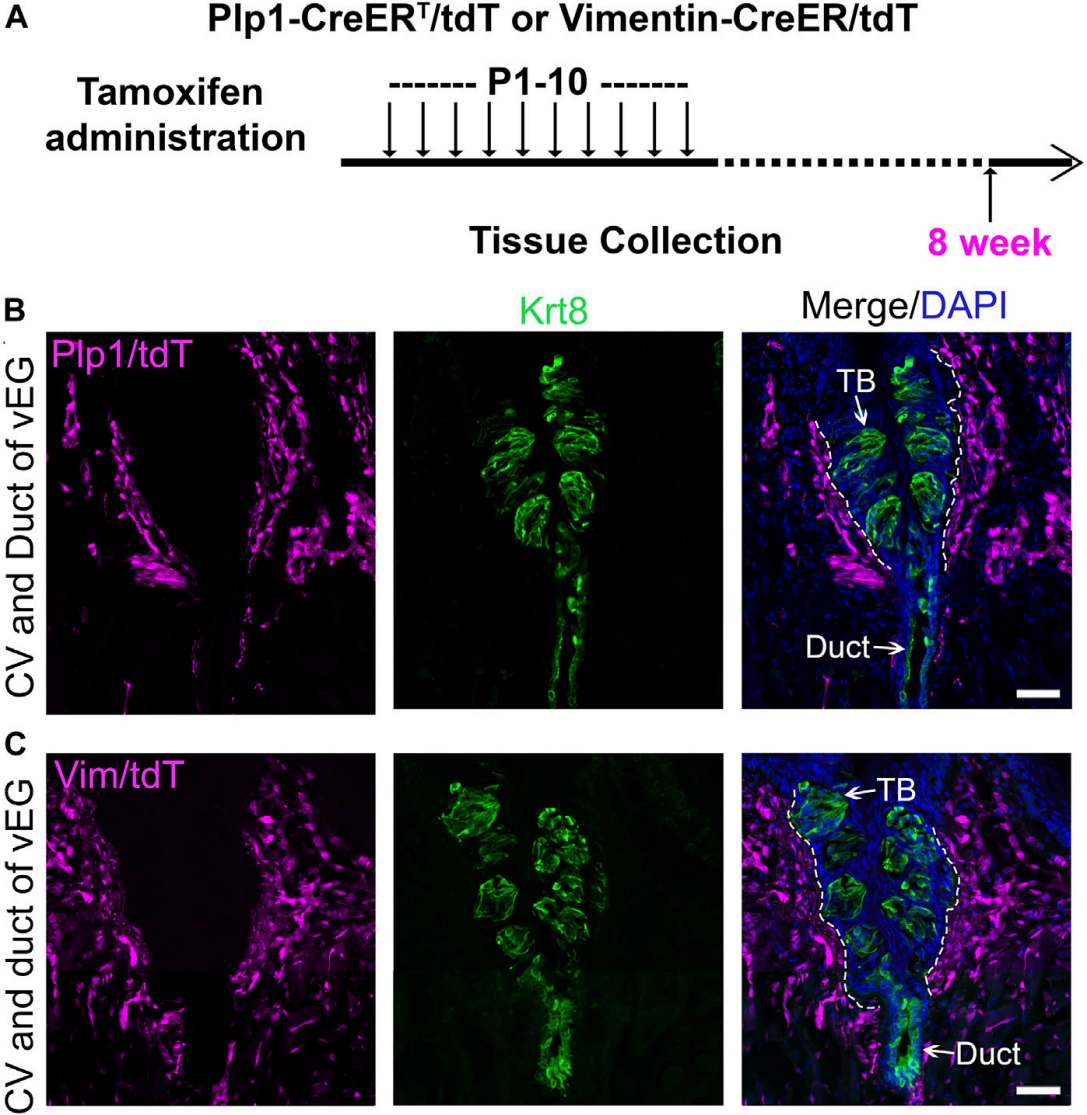
*Plp1-CreER* ^*T*^ */tdT*-traced Schwann cells and *Vimentin-CreER/tdT*-traced stromal cells do not contribute to circumvallate taste buds. **(A)** A schematic diagram to illustrate the timeline of daily tamoxifen administration from P1-10 and tissue collection at 8 weeks of age. **(B,C)**: Single-plane laser scanning confocal images of circumvallate papilla and von Ebner’s gland on coronal sections of *Plp1-CreER* ^*T*^ */tdT* **(B)** and *Vimentin-CreER/tdT* **(C)** mice. tdT^+^ (magenta) are present in the connective tissue core but not in taste buds of circumvallate papilla. Arrows point to Krt8^+^ immunostained (green) cells in taste buds (TB), ductal (Duct) cells of von Ebner’s gland. White dashed lines mark the borders between the epithelium and surrounding connective tissue. Scale bars: 50 μm for all images.

After a long time period post-tamoxifen treatments of ten daily doses of tamoxifen from P1 to P10 (Figure 5A), both *Plp1-CreER* ^*T*^ */tdT* and *Vimentin-CreER/tdT* mice showed sustained extensive distribution of tdT^+^ cells in the connective tissue core of the circumvallate papilla (Figures 5B, C). No tdT^+^ cells were observed in any of the taste buds in the serial sections (Figures 5B, C). Of note, tdT^+^ cells were also absent in the vEG ducts (Figures 5B, C) and acini (Supplementary Figures 5D, 5E). In addition, taste bud-surrounding tongue epithelium was free from tdT tracing signals in *Plp1-CreERT/tdT* and *Vimentin-CreER/tdT* mice at all stages examined (Figures 4B–D, 5B–E).

### The ducts of vEGs express receptors for multiple types of pathogens

**FIGURE 6:**
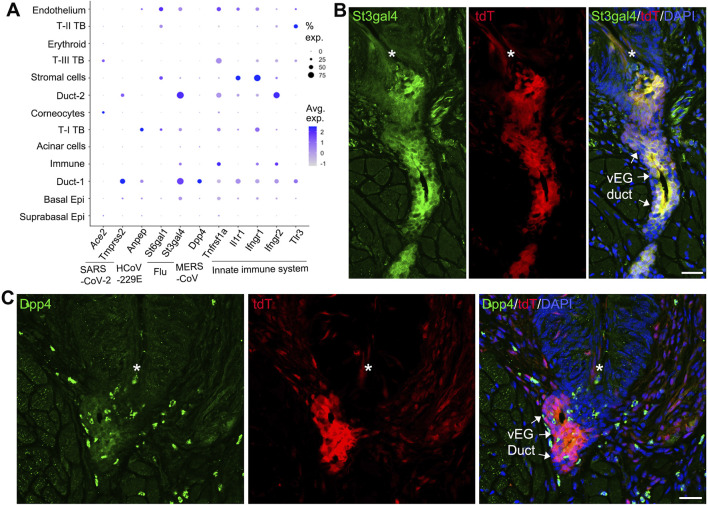
Expression of pathogen receptors in vEG ducts. **(A)** Dot plot to illustrate gene expression [log (UMIs count/10000 + 1)] across the 13 cell clusters in the scRNA-seq data set. Genes related to SARS-CoV-2, HCoV-229E, influenza (Flu), MERS-CoV, and the innate immune system are included. The size of the dots represents the proportion of gene-expressing cells, and the color intensity of the dots represents the average level of the gene expression. **(B,C)** Single-plane laser scanning confocal photomicrographs of coronal sections of circumvallate papilla to demonstrate the distribution of St3gal4^+^ **(B)** and Dpp4^+^ **(C)** cells in *Sox10-Cre/tdT* mice. Asterisks mark the trough of circumvallate trenches. Arrows point the ducts of vEGs labeled by tdT signals. Scale bars: 25 μm.

We have reported the distinct tropism of multiple viruses in the anterior tongue epithelial cells (Wang et al., 2020). In this study, the posterior distribution of viral receptor expression was performed to understand the vulnerability of different cell types in the circumvallate papilla/vEG epithelium to infectious diseases (Figure 6A). We found that different pathogen receptors were enriched in distinct cell clusters. Across all 13 identified cell clusters, the *Sox10*-enriching Duct-1 of vEGs has an overall high frequency in expressing key viral entry factors for SARS-CoV-2 (*Tmprss2*), HCoV-229E (*Anpep*), influenza virus (*St3gal4*), and MERS-CoV45 (*Dpp4* and *Tnfrsf1a*), and multiple receptors involved in the innate immune system. Similarly, the Duct-2 of vEGs enriches multiple pathogen receptors. In contrast, basal tongue epithelial cells (i.e., the well-known taste bud progenitor) depicted a low frequency and abundance of pathogen receptor gene expression. Differentiated taste bud cells showed distinct and relatively low frequency of expression of pathogen receptors.

To validate the pathogen receptor expression from scRNA-Seq, selected receptors (St3gal4 and Dpp4) were detected using immunohistochemistry in the circumvallate papilla/vEG area. We found that St3gal4 immunosignals were highly co-localized with *Sox10-Cre/tdT* signals in the vEG ducts (Figure 6B), which is consistent with the scRNA-Seq data showing the expression of *St3gal4* in Duct-1 and Duct-2 of vEGs. In addition, St3gal4 immunosignals were also seen in the fiber-like structures under the circumvallate papilla trenches. Dpp4 immunostained cells were distributed in the *Sox10-Cre/tdT* ^+^ vEG duct opening region (most likely Duct-1 from scRNA-Seq data) (Figure 6C). Dpp4 cells were also scattered in the connect tissue under the circumvallate papilla.

## Discussion

### *Sox10* ^+^ cells in von Ebner’s glands (vEGs), but not those in connective tissue, serve as progenitors for circumvallate taste buds

Our recent studies have shown that cells expressing *Sox10* (*Sox10* ^+^) represent a novel source of taste bud progenitors that give rise to mainly type-III neuronal taste cells responsible for sour and salt taste (Yu et al., 2020). Three tissue compartments that contain *Sox10* ^+^ cells include the neural crest in mouse embryos, connective tissue core of taste papillae and vEGs in postnatal mice (Yu et al., 2020). Specific mapping of neural crest cell lineage by administering a solitary dose of tamoxifen to *Sox10-iCreER* ^*T2*^ */tdT* ^Tmx@E7.5^ mice resulted in marking of up to 80% of connective tissue cells (neural crest-derived) in the core of the circumvallate papilla. However, this did not trace taste bud cells (Yu et al., 2021a). As a result, it remains uncertain whether *Sox10* ^+^ connective tissue cells are the progenitors of taste buds and whether *Sox10-iCreER* ^*T2*^ */tdT* ^Tmx@E7.5^ marks all neural crest cells. Further efforts were made to map lineages of the two abundant cell subpopulations (i.e., *Plp1* ^+^ Schwann and *Vimentin* ^+^ stromal cells) in the connective tissue core of taste papillae. Our results indicate that the traced cells are not distributed in taste buds. These data are compelling to support that taste buds do not originate from connective tissue cells, The observations in our previous study using *Vimentin-CreER*-driven membrane-bound GFP reporter (Troeger et al., 2012), in which mGFP signals were seen in taste buds (Boggs et al., 2016), may be attributed to the extended nerve fiber or fibroblast lamellipodia from connective tissue.

Another candidate of *Sox10* ^+^ taste bud progenitors is vEGs (Yu et al., 2020) -- the minor salivary glands that are connected to the bottom of the trenches of circumvallate and foliate papillae in the posterior tongue (Riva et al., 1990; Piludu et al., 2006). Indeed, using inducible CreER mouse models under the control of the *Sox10* promoter to trace cells in distinct tissue compartments, the distribution of traced cells in circumvallate taste buds appear concurrently with that in vEGs, but not with that in the connective tissue core of circumvallate papilla.

**FIGURE 7:**
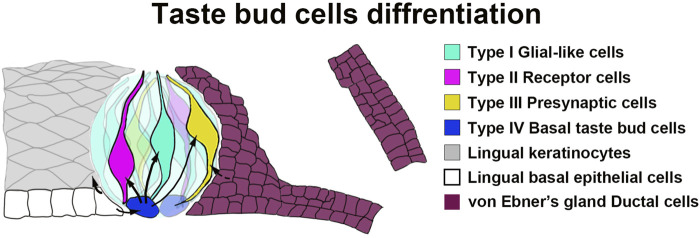
A schematic diagram illustrating the tissue compartments contributing to circumvallate taste buds. Our data indicate that *Sox10*-expressing cells in von Ebner’s gland serve as stem/progenitor cells for taste bud cells in the circumvallate papilla.

Of note, considering the importance of proper use of CreER and Cre reporter mouse lines, the concurrent distribution of traced cells in vEGs with those in circumvallate taste buds was confirmed with two different Cre reporters and two independently developed CreER under the control of *Sox10* promoter (*Sox10-iCreER* ^*T2*^ */tdT* and *Sox10-CreER* ^*T2*^ */YFP*). Together with the previous observations that taste buds exist in the circular structure of tissue transplants in the deep layer under the circumvallate papilla trench (Zalewski, 1976), our data demonstrate that vEGs are the source of *Sox10* ^+^ progenitors for taste buds (Figure 7).

### vEG ducts are the source of *Sox10* ^+^ taste bud progenitors

The vEGs are one type of the minor salivary glands that are connected and open to the trough of circumvallate and foliate taste papillae (Hand et al., 1999). Minor salivary glands are structurally comprised of the endpieces called acini and the ducts including interlobular, intercalated, excretory and collecting (main) ducts. In mice, the vEGs at vallate papilla are located under circumvallate taste buds and composed of serous acini and interlobular excretory ducts (Chen et al., 2023). At the opening of vEG duct (main duct) the columnar epithelium transitions to stratified squamous epithelium (Hand et al., 1999; Chen et al., 2023).

Our scRNA-seq data in circumvallate papilla/vEG complex from *Sox10-Cre/tdT* mice allows us to identify the heterogeneity of epithelial cell population and to compare the *Sox10* ^+^ and tdT^+^ traced cells at the single cell resolution. The enrichment of *Sox10* expression in one of vEG ductal cell clusters (Duct-1) contrasts that of *tdT* ^*+*^ signals (marking the *Sox10* ^*+*^ cell lineage) in the duct and additional cells in its immediate neighbor tissues, i.e., type-III taste bud cells and Duct-2, indicating that *Sox10* ^*+*^ vEG Duct-1 give rise to Duct-2 and type-III taste bud cells. This is also supported by the cell trajectory analysis, co-localization of *Sox10-iCreER* ^*T2*^ */tdT* signals and type-III taste cell marker SNAP25, and abundant distribution of proliferating cells in vEG ducts. Based on our observations of the abundant distribution of proliferating cells in the main ducts of vEGs, we speculate that *Sox10* ^*+*^ vEG Duct-1 is the main duct. Because different cell types may share common molecular markers (Aure et al., 2015; Rocchi et al., 2021; Chen et al., 2022), it is challenging to locate the taste bud progenitors in vEGs based on the scRNA-Seq data. Combined information of the spatial distribution, cell morphology and molecular signatures is necessary to pinpoint the taste bud progenitors in vEGs. Further studies are needed to verify the location(s) and cell type(s) of *Sox10* ^*+*^ taste bud progenitors in the vEG ducts.

Regarding the derivation of other types of taste cells, both *Sox10* and *tdT* expression was enriched in the same small percentage of type-I cells suggesting that the *Sox10-Cre/tdT*-traced signals (Yu et al., 2020) were due to the *Sox10* expression in the type-I cells. In this study, we did not detect *tdT* expression in type-II taste cells. A large-scale scRNA-Seq study may be needed to identify a low number, if any, of traced type-II taste cells.

*Lgr5* is a known taste progenitor marker for taste buds (Takeda et al., 2013; Yee et al., 2013; Ren et al., 2014). It was recently reported that *Lgr5* ^+^ cell lineage was mapped in the vEGs (Defoe et al., 2024). Our scRNA-Seq data revealed the expression of *Lgr5* in basal tongue epithelial cells but not in the vEGs. Likely the *Lgr5*-traced vEGs are derived from the basal tongue epithelial cells as it has been reported that the circumvallate trenches develop first and then invaginate and gives rise to vEGs (Lee et al., 2006; Kim et al., 2009). Thus, *Sox10* ^+^ taste progenitors in vEG ducts represent a distinct population of taste progenitors from those marked by *Lgr5*. Further studies are needed to distinguish the *Sox10* ^+^ and *Lgr5* ^+^ taste progenitors in the circumvallate/vEG complex.

While *Sox10-CreER* traced cells are present in both circumvallate taste buds and vEGs, no traced cells are found in the lingual epithelium surrounding taste buds. This suggests that the ductal cells of Ebner’s glands migrate and differentiate directly into taste bud cells, rather than being “transited” by becoming the taste bud-surrounding lingual epithelial cells. It has been recently reported that *Nkx2-2* ^+^ taste cells are committed to the type-III lineage (Qin et al., 2021). *Sox10* ^+^ vEG ductal progenitors may initiate the expression of *Nkx2-2* for the terminal differentiation to type-III taste cells. Overall, our data add vEG ducts to the progenitor sources for circumvallate taste buds (Figure 7).

### vEG ductal taste bud progenitors express *Sox10* transiently and are susceptible to pathogen infections

When comparing the abundance of both *Sox10-iCreER* ^*T2*^ */tdT* and *Sox10-CreER* ^*T2*^ */YFP*-traced cells in circumvallate taste buds after a short vs. long term tamoxifen administration, our data reveal one of the features of *Sox10* ^+^ vEG taste bud progenitors. After inducing Cre recombination in neonatal mice for 10 days, *Sox10-CreER* efficiently labeled vEGs, but only a limited number of cells were traced in the circumvallate taste buds several weeks later. A prolonged series of tamoxifen treatments lead to a dramatically increased number of traced taste bud cells. The data suggests a transient *Sox10* expression or a short lifespan of *Sox10* ^+^ vEG taste bud progenitors.

Another feature of the *Sox10* ^+^ vEG taste bud progenitors is the enriched expression of pathogen receptors. In our previous study (Wang et al., 2020), scRNA-Seq profiling of anterior mouse tongue epithelial cells (Schaum et al., 2018) revealed that molecules for various viruses’ entries into cells are enriched in different cell clusters. Our current scRNA-Seq data from the circumvallate/vEG complex reveals distinct tropism of multiple viruses in the posterior tongue region in a different pattern from that in the anterior tongue. Combined data in both anterior and posterior tongue epithelium provide insights into the mechanisms underlying taste loss in different infectious diseases. For example, in taste cells (type-III) we detected SARS-CoV-2 receptor gene *Ace2*; in taste bud progenitors such as *Sox10* ^+^ vEG ducts and in the taste bud-surrounding epithelial cells we detected *Ace2* and co-receptor *Tmprss2*. We acknowledge that mouse ACE2 is, unlike humans, not susceptible to SARS-CoV-2 (Zhou et al., 2020); however, given that mice and humans share similar gene expression patterns. If it is true that ACE2 and TMPRSS2 are expressed in taste cells and taste bud progenitors in the posterior tongue region of humans, the data suggests that direct SARS-CoV-2 infection of taste cells may be the cause of acute taste loss, and the infection of taste bud progenitors may cause a sequela, i.e., long-term taste dysfunction. As the newly found taste bud progenitor source, *Sox10* ^+^ vEG ducts enrich expressing genes encoding multiple pathogen receptors making the organ susceptible to infections.

### Derivation of connective tissue cells in the tongue

The tongue is a complex and highly organized organ in which the mesenchymal/connective tissue cells serve as scaffolds to compartmentalize different tissues (Chai and Maxson, 2006; Cordero et al., 2011). In the connective tissue core of taste papillae, we observed two profuse and largely distinct subpopulations of cells that express Vimentin, an intermediate filament protein widely used as a marker for mesenchymal stromal cells (Hol and Capetanaki, 2017) and Proteolipid protein 1 (Plp1), an intrinsic membrane protein specifically expressed in Schwann cells in the peripheral nervous system (Kamholz et al., 1992). The cells in the tongue mesenchyme/connective tissue are largely derived from the cranial neural crest (O’Rahilly and Müller, 2007; Thirumangalathu et al., 2009; Liu et al., 2012; Parada et al., 2012; Boggs et al., 2016; Yu et al., 2020; Yu et al., 2021a). Multiple mouse models have been used to map neural crest lineages in the tongue, including *Wnt1-Cre* (Chai et al., 2000; Jiang et al., 2002; Ito et al., 2003; Tallquist and Soriano, 2003; Brewer et al., 2004; Merrill et al., 2006; Yoshida et al., 2008; Katayama et al., 2011), *P0-Cre* (Liu et al., 2012), *Dermo1-Cre* (Geske et al., 2008; Cornett et al., 2013; Boggs et al., 2016), and *Sox10-Cre* (Matsuoka et al., 2005; Hari et al., 2012). The Cre models label cells in the tongue mesenchyme of embryos and connective tissue in the postnatal mouse tongue (Boggs et al., 2016; Yu et al., 2020). However, none of these models labels neural crest cells exclusively or labels all neural crest cells (Chen et al., 2017; Debbache et al., 2018). There is no available data regarding the proportion of neural crest- and non-neural crest-derived cells.

*Sox10* is specifically expressed in the neural crest in early embryos (Southard-Smith et al., 1998; Yu et al., 2020). Inducible *Sox10-CreER* (Yu et al., 2021a) with tamoxifen administration at E7.5 (Ved et al., 2019; Sun et al., 2021) is optimal in mapping neural crest cell lineages exclusively. A single-dose tamoxifen administration to the pregnant dam carrying E7.5 *Sox10-iCreER* ^*T2*^ */tdT* embryos results in extensive labeling of tongue mesenchymal cells at E12.5 (Yu et al., 2021a). In the present study, quantification of *Sox10-iCreER* ^*T2*^ */tdT* traced cells in the connective tissue core of circumvallate papilla indicates that at least 80% of the cells are neural-crest derived. An increase in tamoxifen dose(s) may lead to a higher proportion of labeled cells; however, embryonic lethality limited the study. To confirm if up to 20% of connective tissue cells in the tongue originate from mesoderm, specific mouse models need to be used for analyses.

## Conclusion

Together with the previous findings (Zalewski, 1976; Yu et al., 2021a), our data in the present study supports the concept that *Sox10* ^+^ cells in the ducts of vEGs can migrate and differentiate to circumvallate taste bud cells that are mainly type-III for sour and salt (Figure 7). These *Sox10* ^+^ cells represent a novel source of taste bud progenitors in distinction from the basal lingual epithelial cells. The differentiation of Ebner’s gland cells into taste bud cells for transducing gustatory signals represents a novel format of tissue-tissue interactions in the circumvallate/vEG complex in addition to gland’s secreting saliva to facilitate taste perception.

## Materials and methods

### Animals

Mice were used in this study (Table 1). The animal use was approved by the Institutional Animal Care and Use Committee at The University of Georgia and the Medical University of Vienna and complied with the National Institutes of Health Guidelines for the care and use of animals for research.

**TABLE 1:** Mouse strains that were used.

Strains	Sources
C57BL/6J Wild type	The Jackson Laboratory (Stock#000664)
FVB/NJ wild-type	The Jackson Laboratory (Stock#001800)
*Plp1-CreER* ^*T*^	The Jackson Laboratory (Stock#024701) (Doerflinger et al., 2003)
*Plp1-CreER* ^*T2*^	U Suter lab at ETH Zurich, Switzerland (MGI ID #2663093) (Leone et al., 2003; Kameneva et al., 2021a; Kameneva et al., 2021b)
R26-tdTomato	The Jackson Laboratory (Stock #007914) (Madisen et al., 2010)
*Sox10-Cre*	The Jackson Laboratory (Stock#025807) (Matsuoka et al., 2005)
*Sox10-iCreER* ^*T2*^	The Jackson Laboratory (Stock#027651) (McKenzie et al., 2014)
*Sox10-CreER* ^*T2*^	Vassilis Pachnis lab at Crick (MGI ID #5301107) (Laranjeira et al., 2011; Kameneva et al., 2021a)
Vimentin-CreER	Robert F Schwabe lab in Columbia University (Troeger et al., 2012)
YFP	The Jackson Laboratory (Stock #006148) (Srinivas et al., 2001)

Mice were maintained in the animal facility of the Animal and Dairy Science department at the University of Georgia at 22°C under 12-h day/night cycles. *Sox10-Cre* (Matsuoka et al., 2005), *Sox10-iCreER* ^*T2*^ (McKenzie et al., 2014), *Plp1-CreER* ^*T*^ (Doerflinger et al., 2003), and *Vimentin-CreER* (Troeger et al., 2012) mice were bred with *R26-tdTomato* (tdT) Cre reporter mice (Madisen et al., 2010) respectively to generate *Sox10-Cre/tdT, Sox10-iCreER* ^*T2*^ */tdT*, *Plp1-CreER* ^*T*^ */tdT*, and *Vimentin-CreER/tdT* mice. Being aware of the importance of use of proper CreER and reporter mouse lines, two independent CreER [*Plp1-CreER* ^*T2*^ (Leone et al., 2003; Kameneva et al., 2021a; Kameneva et al., 2021b) and *Sox10-CreER* ^*T2*^ (Laranjeira et al., 2011; Kameneva et al., 2021a)] mouse lines crossed with a different Cre reporter *YFP* (yellow fluorescent protein) (Srinivas et al., 2001) were used to verify our observations. The posterior tongues containing circumvallate taste buds and von Ebner’s glands were provided by Dr. Igor Adameyko’s lab at the Medical University of Vienna. Data from female and male mice did not show significant differences regarding the distribution of labeled cells in each strain; therefore, females and males were grouped for analyses. FVB/NJ wild-type mice (The Jackson Laboratory, Stock#001800) were used to breed in parallel for fostering cesarean-born *Sox10-iCreER* ^*T2*^ */tdT* pups. Polymerase chain reaction (PCR) genotyping was performed using the following primers listed in Table 2.

**TABLE 2:** PCR genotyping primers.

Targets	Purposes	Forward sequences	Reverse sequences
CreER	Mouse CreER and CreER^T^ recombinase allele	5′-ATT GCT GTC ACT TGG TCG GC-3′	5′-GGA AAA TGC TTC TGT CCG TTT GC-3′
iCreER^T2^	Mouse iCreER^T2^ recombinase allele	5′-GAG ACG GAC CAA AGC CAC T-3′	5′-CTG CAG CCT CCA CTG-3′
msSRYz_SexDet	The sex of mouse embryos	5′-TTG TCT AGA GAG CAT GGA GGG CCA TGT CAA-3′	5′-CCA CTC TGT GAC ACT TTA GCC CTC CGA-3′
tdT	Expression of tdT reporter	5′-CTG TTC CTG TAC GGC ATG G-3′	5′-GGC ATT AAA GCA GCG TAT CC-3′

### Tamoxifen administration and fostering of pups

Tamoxifen (Tmx) (Cat No. T5648; Sigma-Aldrich, Inc., St. Louis, MO) was dissolved in the mixture of ethanol and corn oil (1:9) at the working concentration of 10.0 or 11.1 mg/mL. CreER-mediated DNA recombination was induced by oral gavage of 0.1 mL tamoxifen solution to the pregnant or nursing dams. Vehicle controls were treated with corn oil in the dams or littermates with the same genotype in parallel.

For tracing neural crest cell lineages, timed pregnant mice were used. Noon of the day of vaginal plug detection in mice was designated as embryonic day (E) 0.5. To induce Cre recombination in neural crest cells, a single dose of 0.1 mL tamoxifen solution was given to the pregnant dams that carried E7.5 *Sox10-iCreER* ^*T2*^ */tdT* embryos through oral gavage using 16 G × 38 mm polyurethane feeding tubes (Cat No. FTPU-16-50, Instech Laboratories, Inc., Plymouth Meeting, PA). To solve the dystocia problem caused by tamoxifen treatment in pregnant mice, cesarean sections were performed to deliver the *Sox10-iCreER* ^*T2*^ */tdT* embryos at E18.5. The delivered pups were fostered by FVB/NJ nursing dams immediately after cesarean birth.

To activate CreER-induced recombination in neonatal mice, nursing dams were given 0.1 mL tamoxifen daily from birth (P1) to P10. The nursing dams for *Sox10-CreER* ^*T2*^ */YFP* pups received 0.1 mL tamoxifen every other day. For mice with prolonged tamoxifen administration, 0.1 mL tamoxifen solution was given to the nursing dams once every three days until weaning on day 28, followed by oral gavage to weaned mice once every three days until 8 weeks or 16 weeks.

### Tissue collection

Postnatal (P) mice were harvested on day 11, week 8 or 16 of age (n = 3 for both vehicles- and tamoxifen-treated at each stage). Mice were euthanized with CO_2_ followed by cervical dislocation. For scRNA-Seq, the tongue was immediately dissected for cell dissociation as previously described (Yu et al., 2021b). For immunohistochemistry on tissue sections, transcardial perfusion was performed using 10 mL warm 0.1 M PBS, 10 mL warm, and 20 mL cold 2% PFA in 0.1 M PBS. Tongues were dissected and further fixed in 2% PFA at 4°C for 2 h.

### Single cell RNA-sequencing (*scRNA*-seq)

*SOX10-Cre/tdT* mice were used at 8 weeks of age when the proportion of traced cells in circumvallate taste buds is abundant and plateaued (Yu et al., 2020). The separation of the epithelium of circumvallate papilla region and cell dissociation were performed as previously described (Yu et al., 2021b). A total of 8 circumvallate/vEG epithelial sheets were pooled for single cell isolation. Dissociated cells (for a target of sequencing ∼10,000 cells) of circumvallate papilla/vEG epithelium were submitted to the Univ of Georgia–Georgia Genomics and Bioinformatics Core for scRNA-seq library preparation (10X Genomics) and Illumina sequencing.

The reads from sequencing were processed using Cell Ranger (v7.0.0) for alignment, filtering, barcode counting, UMI counting and cell counting. A customized reference genome was built by adding gene tdTomato (tdT) to mouse reference genome (mm10). Analysis of scRNA-seq dataset was performed with R package Seurat (v5.0.1). We followed the analysis pipeline recommended by Satija Lab (https://satijalab.org). In cell quality control, cells with less than 500 total RNA counts and less than 250 detected features were excluded. Additionally, cells with mitochondrial gene exceeding 20% were filtered out to remove potentially stressed or dying cells. Data was normalized and PCAs were calculated using “RunPCA,” Uniform Manifold Approximation and Projection (UMAP) analyses have been done using “RunUMAP” (dims = 1:20). “FindNeighbours” was used for defining the distance between the cells and later these were grouped using “FindClusters” with resolution 0.1. Subsequently, clusters were annotated using marker genes from the literature. For trajectory analysis, Monocle3 (v1.3.4) was employed, following the recommended analysis pipeline by Trapnell Lab (https://cole-trapnell-lab.github.io/monocle3/) (Trapnell et al., 2014; Qiu et al., 2017; Cao et al., 2019).

### Immunohistochemistry

Fixed tongues were cryoprotected in 30% sucrose in 0.1 M PBS for at least 48 h at 4°C. The circumvallate papilla was dissected from the tongues and trimmed under a stereomicroscope, embedded in O.C.T. (Cat. No. 23-730-571, Thermo Fisher Scientific, Pittsburgh, PA), and rapidly frozen. Coronal sections were cut at 8 μm in thickness and mounted onto charged slides (Fisher brand™ Superfrost™ Plus Microscope Slides, Cat. No. 12-550-15, Thermo Fisher Scientific, Pittsburgh, PA). Non-specific binding was blocked with 10% normal donkey serum (Cat. No. SLBW 2097, Sigma-Aldrich, Inc., St. Louis, MO) in 0.1 M PBS containing 0.3% Triton X-100 (Cat. No. X100-100ML, Sigma-Aldrich, Inc., St. Louis, MO) for 30 min, followed by overnight incubation with primary antibody against GFP (1:500, SKU: GFP-1020, Aves Labs), or Keratin (Krt) 8 (1:500, TROMA-1, Developmental Studies Hybridoma Bank), or Ki67 (1:200, ab16667, ABCAM, MA), or Plp1 (1:500, #28702, Cell Signaling Technology, Danvers, MA), or DPP4 (1:200, MA5-32643, Invitrogen), or ST3GAL4 (1:200, 13546-1-AP, Invitrogen) diluted with 0.1 M PBS containing 0.3% Triton X-100% and 1% normal donkey serum. After rinses in 0.1 M PBS (3 times, 10 min each), sections were incubated with Alexa Fluor^®^ 647 (Krt8) secondary antibody (1:500, Invitrogen, Eugene, OR) and Alexa Fluor^®^ 488 (GFP, Ki67, Plp1, DPP4, ST3GAL4) secondary antibody (1:500, Invitrogen, Eugene, OR) for 1 h at room temperature. Sections were rinsed and then counterstained with DAPI (200 ng/mL, Cat. No. D1306, Thermo Fisher Scientific, Pittsburgh, PA) for 10 min at room temperature. After rinses with 0.1 M PBS followed by dipping in Milli-Q water (DirectQ^®^ 3 UV water purification system, Millipore, MA). Sections were air-dried and cover-slipped with Prolong^®^ diamond antifade mounting medium (Cat. No. P36970, Thermo Fisher Scientific, Pittsburgh, PA).

### Photomicroscopy and quantifications

Immunostained sections were thoroughly examined under a fluorescent light microscope (EVOS FL, Thermo Fisher Scientific, Pittsburgh, PA) and images were taken using a laser scanning confocal microscope in the UGA Microscopy Core (Zeiss LSM 710 or 880, Zeiss, Germany). The total cells and *Sox10-iCreER* ^*T2*^ */tdT*-unlabeled cells in connective tissue immediately under circumvallate taste buds were quantified on single-plane confocal images from 8-week-old *Sox10-iCreER* ^*T2*^ */tdT* ^Tmx@E7.5^ mice (n = 3) using Photoshop software. The region of underlying connective tissue within 100 μm in distance to the basement membrane of the circumvallate papilla epithelium was included for the quantification. For each mouse, three sections were selected for analysis, i.e., the section with the deepest trench and biggest area of circumvallate papilla, and those two anterior and posterior sides of sections at 40 μm away from the section with the deepest trench. Only the cells with a clear DAPI^+^ nucleus were counted. A cell with a clear DAPI^+^ nucleus and without any tdT signals in the cell was regarded as a *Sox10-iCreER* ^*T2*^ */tdT* non-labeled cell. The proportion of neural crest-derived connective tissue cells was calculated using the formula of [DAPI^+^ tdT^+^ cells/DAPI^+^ (tdT^−^ + tdT^+^) cells].

## Data Availability

The data presented in the study are deposited in the NCBI GEO (Gene Expression Omnibus) repository, accession number GSE273518.
