# Supplementary material for: The duct of von Ebner’s glands is a source of Sox10 + taste bud progenitors and susceptible to pathogen infections
Source: Front Cell Dev Biol. 2024 Aug 23;12:1460669. doi: 10.3389/fcell.2024.1460669 (PMC11377339; doi:10.3389/fcell.2024.1460669)
Supplement: Supplementary file 1 [file DataSheet1.docx]

## Supplementary Figures

**Supplementary Figure 1.** Cell labeling and lineage mapping of Sox10^+^ cells in von Ebner’s glands. **A:** A schematic diagram to show the experimental paradigm in *Sox10-iCreER^T2^/tdT* mice. **B-C:** Single-plane laser scanning confocal photomicrographs on coronal sections of von Ebner’s glands. Following tamoxifen administration from P1 to P10, *Sox10-iCreER^T2^/tdT^+^* cells (magenta) were abundantly distributed in the ducts and acini of von Ebner’s glands at P11 (B) and 8- weeks (C) of mice. Scale bars: 50 μm in all images.

Supplementary Figure 1


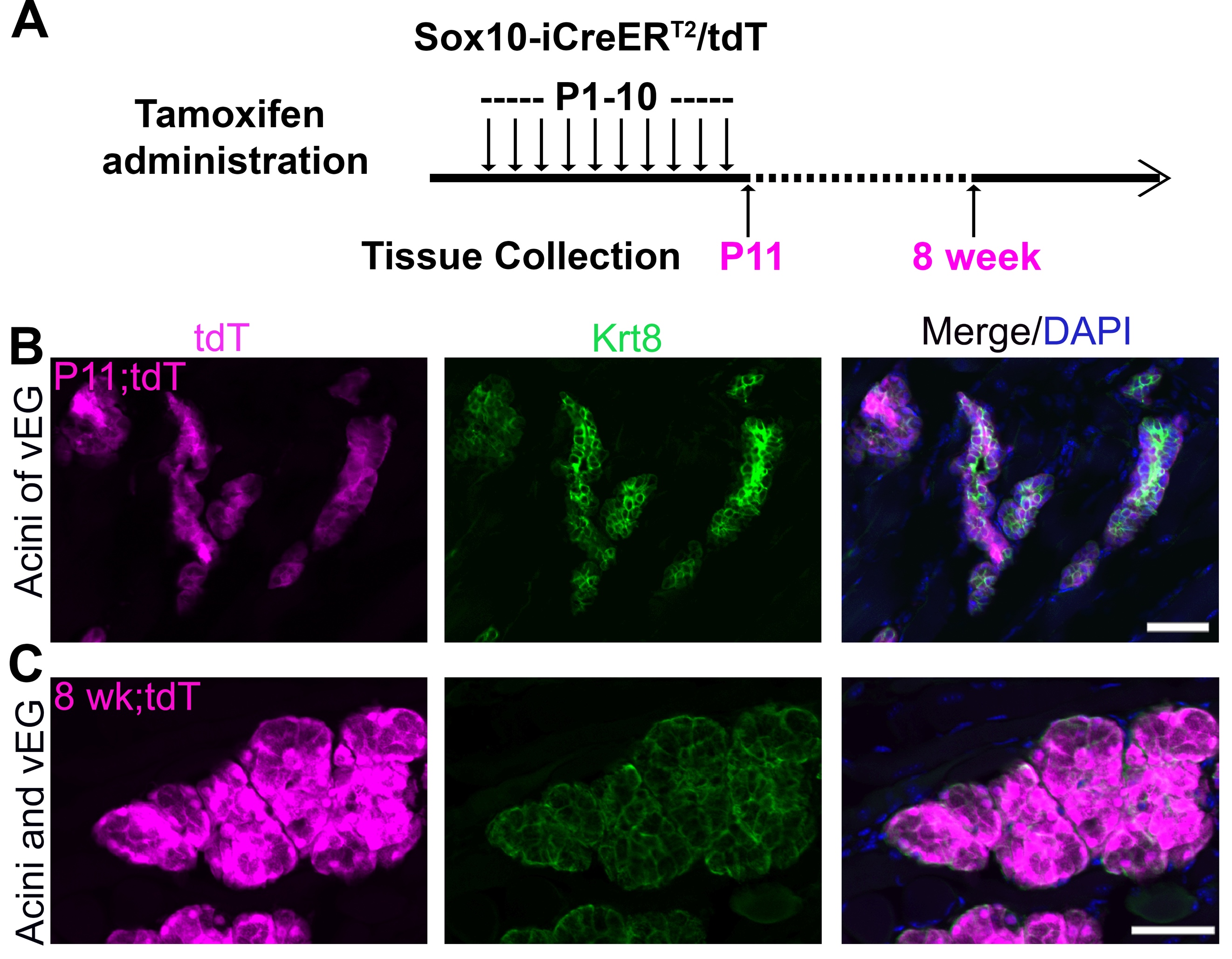


**Supplementary Figure 2.** Abundant distribution of *Sox10-iCreER^T2^/tdT*-labeled cells in the acini of von Ebner’s glands in mice treated with tamoxifen for a long-term (8-wk or 16-wk). **A:** A schematic diagram to illustrate the experimental paradigm for tamoxifen administration and tissue collection from *Sox10-iCreER^T2^/tdT* mice at 8 or 16 weeks. **B**-**C:** Single-plane laser scanning confocal images of von Ebner’s glands on coronal sections of circumvallate papilla region in 8-week-old (B) and 16-week-old (C) mice. *Sox10-iCreER^T2^/tdT*^+^ cells (magenta) are abundantly distributed in the acini of von Ebner’s glands. Scale bars: 50 μm in all images.

Supplementary Figure 2


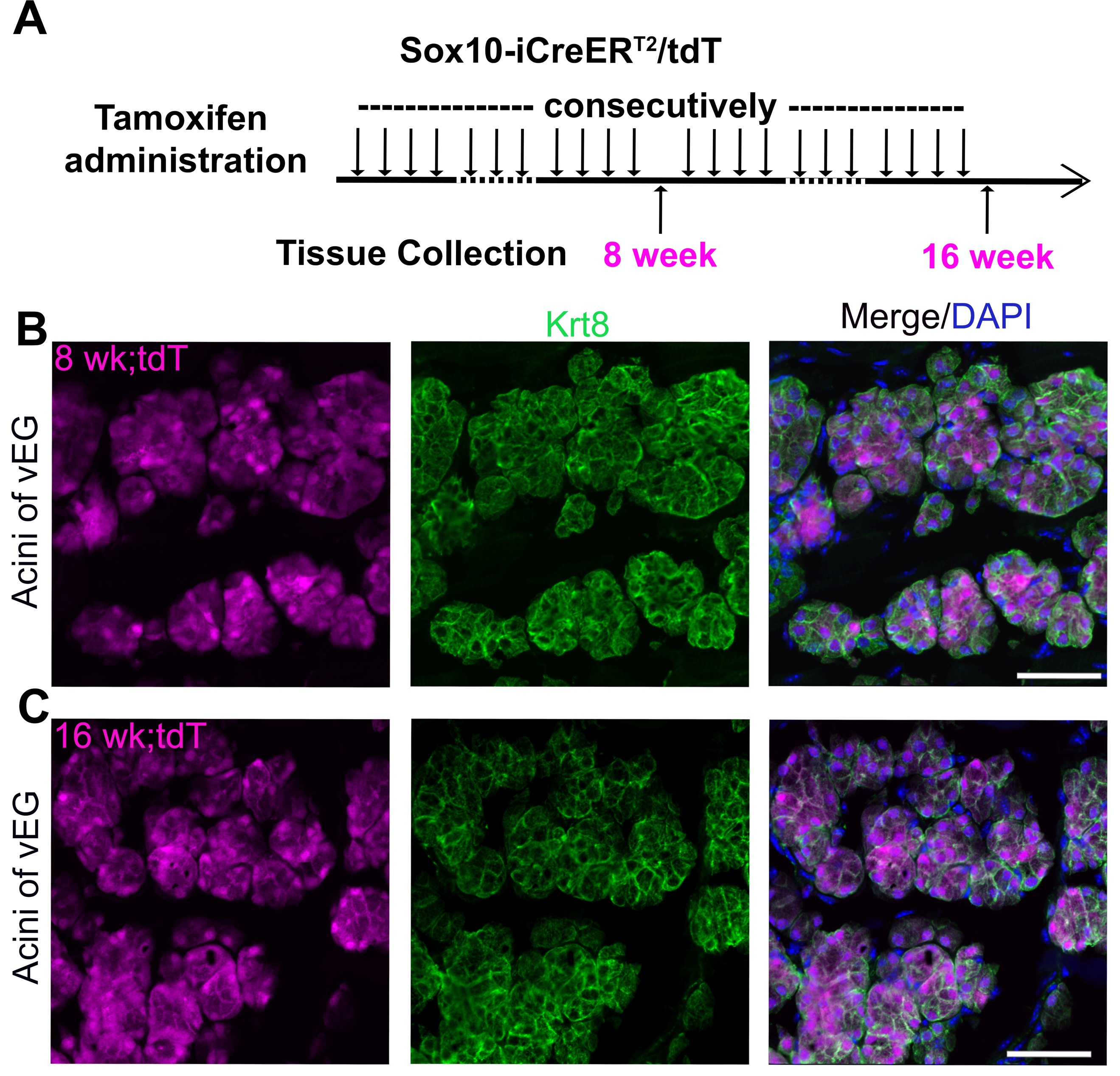


Supplementary Figure 3


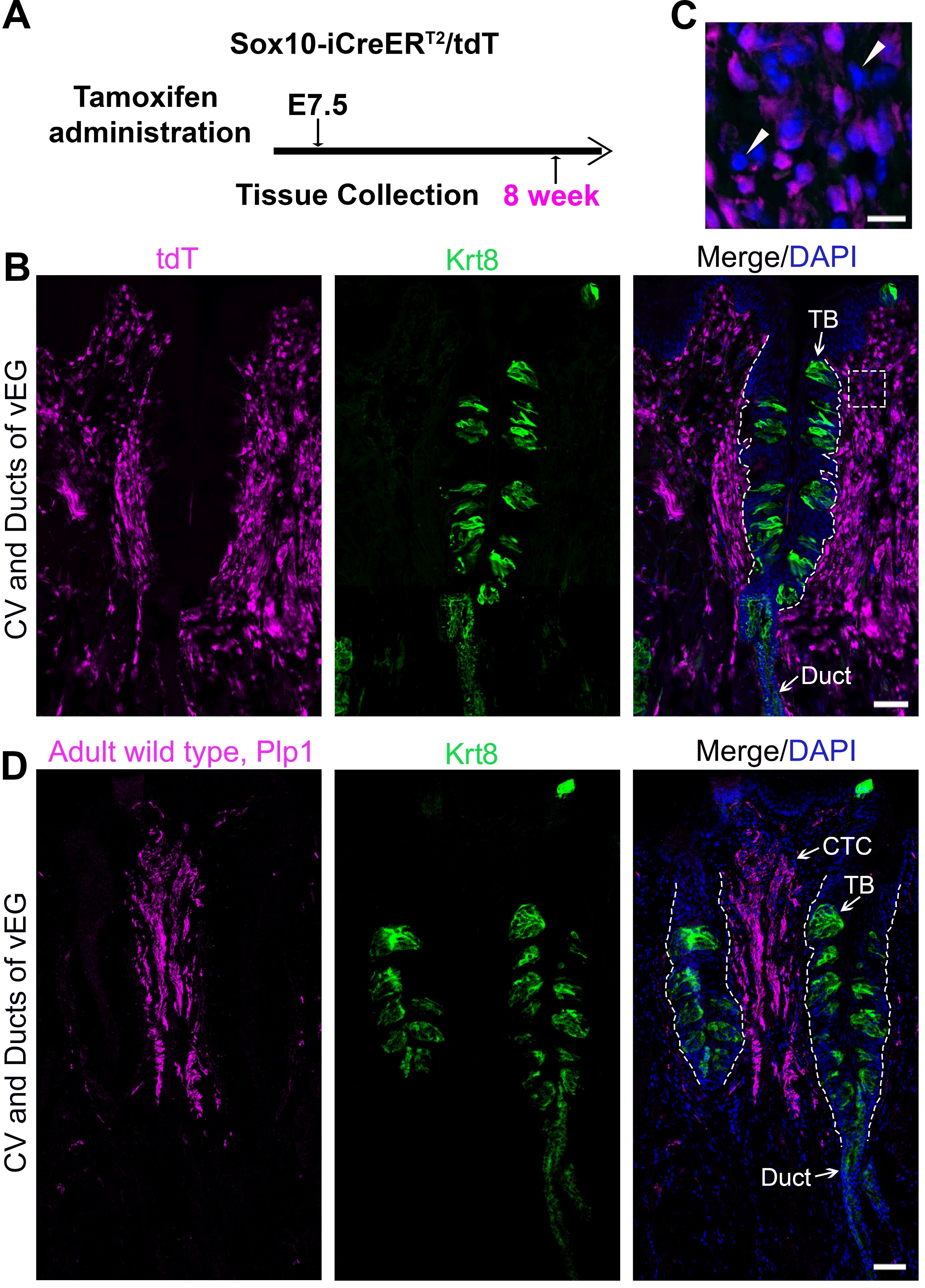


**Supplementary Figure 3.** The distribution of neural crest-derived cells and Plp1-expressing (Plp1^+^) cells in the connective tissue core of circumvallate papilla. **A:** A schematic diagram to illustrate the experimental design using *Sox10-iCreER^T2^/tdT* mice for neural crest cell mapping in B-C. **B-D:** Single-plane laser scanning confocal photomicrographs of coronal sections of circumvallate papilla to demonstrate the distribution of *Sox10-iCreER^T2^/tdT^+^*-traced (B-C) and Plp1^+^ immunostained (D) cells (magenta). Image in C is the high magnification of squared area in B. Arrowheads point to Krt8^+^ immunostained (green) cells in taste buds (TB), ductal (Duct) cells of von Ebner’s gland, and connective tissue core (CTC) of circumvallate papilla. Arrowheads in C point to the tdT^-^ cells. White dashed lines in B and D mark the borders between the epithelium and underlying connective tissue. Scale bars: 50 μm in B and D; 10 μm in C.

Supplementary Figure 4


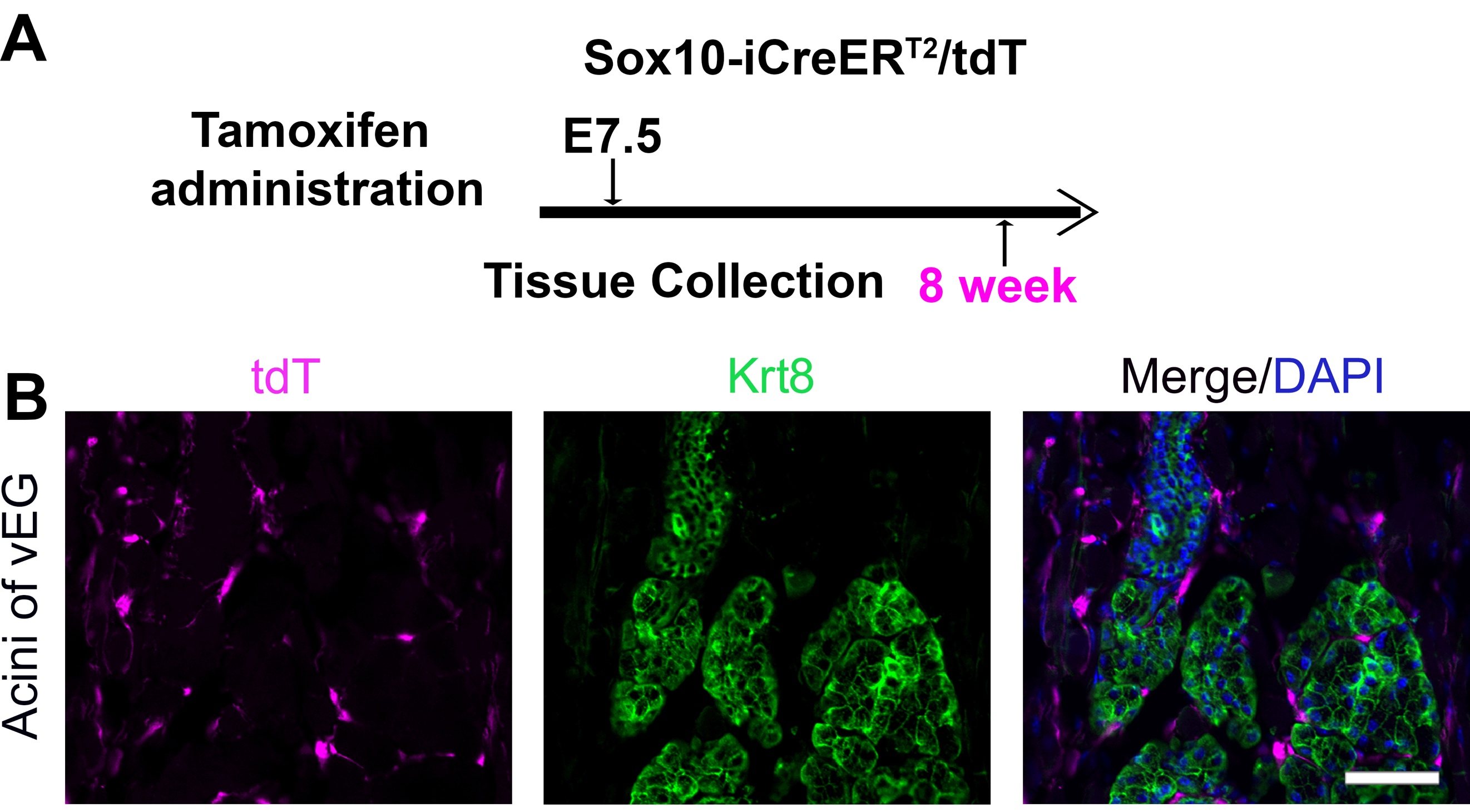


**Supplementary Figure 4.** Neural crest cell lineage mapping in the acini of von Ebner’s glands**. A:** A schematic diagram to illustrate the experimental design using *Sox10-iCreER^T2^/tdT* mice for neural crest cell mapping. **B:** Single-plane laser scanning confocal photomicrographs of von Ebner’s glands on coronal sections. *Sox10-iCreER^T2^/tdT*-labeled cells (magenta) are scattered in the surrounding tissue but not seen in the glands. Scale bar: 50 μm.

Supplementary Figure 5


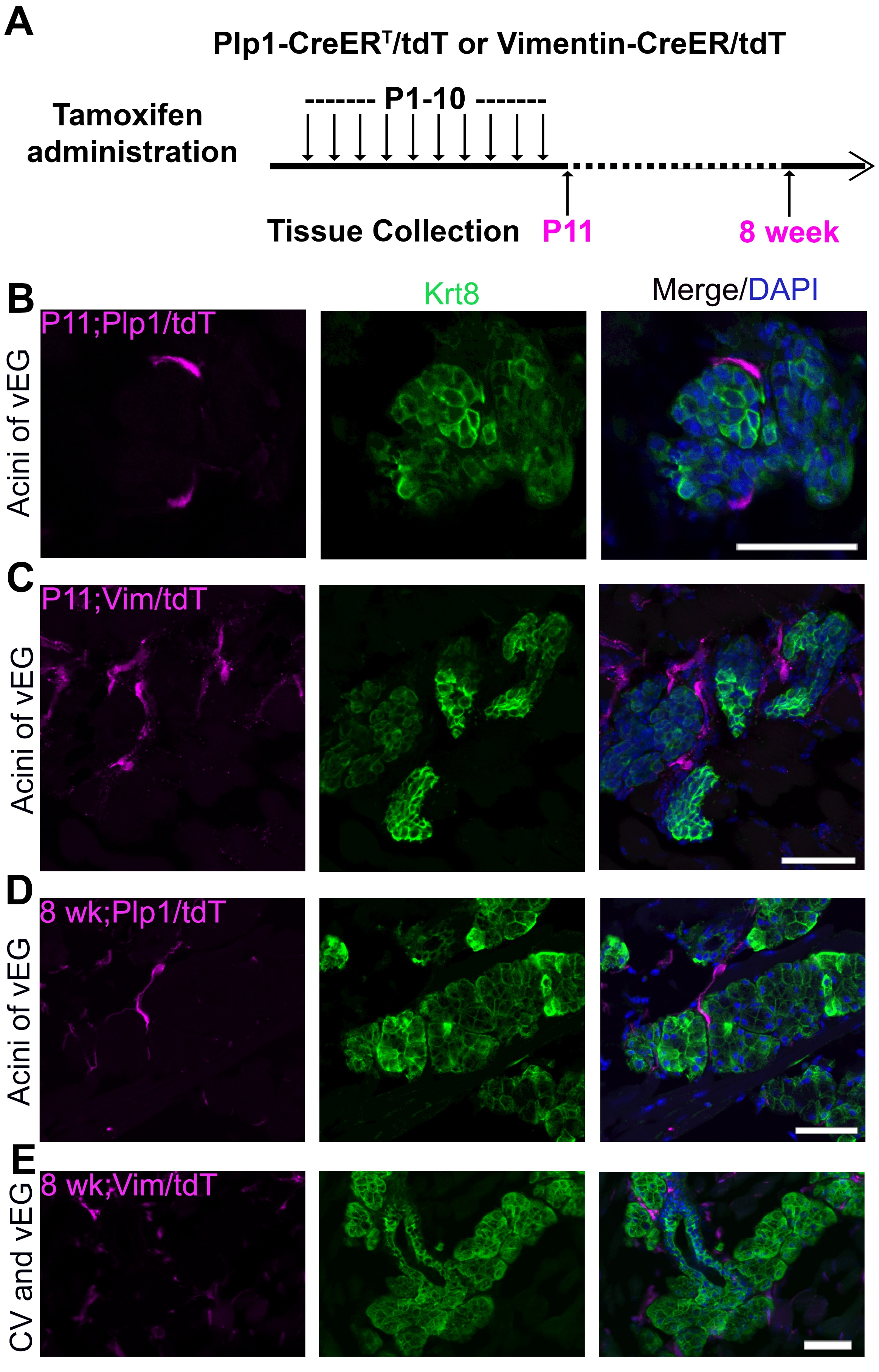


**Supplementary Figure 5.** Cell labeling and lineage mapping in von Ebner’s glands using *Plp1-CreER^T^/tdT* and *Vimentin-CreER/tdT* mice with tamoxifen administration from P1 to P10. **A**: A schematic diagram to illustrate the timeline of daily tamoxifen administration from P1-10 and tissue collection at P11 day and 8 weeks. **B-E**: Single-plane laser scanning confocal images of von Ebner’s glands on coronal sections of circumvallate papilla region in *Plp1-CreER^T^/tdT* (B, D) and *Vimentin-CreER/tdT* (C, E) mice. Scale bars: 50 μm in all images.
